# Atmospheric new particle formation identifier using longitudinal global particle number size distribution data

**DOI:** 10.1038/s41597-024-04079-1

**Published:** 2024-11-16

**Authors:** Simonas Kecorius, Leizel Madueño, Mario Lovric, Nikolina Racic, Maximilian Schwarz, Josef Cyrys, Juan Andrés Casquero-Vera, Lucas Alados-Arboledas, Sébastien Conil, Jean Sciare, Jakub Ondracek, Anna Gannet Hallar, Francisco J. Gómez-Moreno, Raymond Ellul, Adam Kristensson, Mar Sorribas, Nikolaos Kalivitis, Nikolaos Mihalopoulos, Annette Peters, Maria Gini, Konstantinos Eleftheriadis, Stergios Vratolis, Kim Jeongeun, Wolfram Birmili, Benjamin Bergmans, Nina Nikolova, Adelaide Dinoi, Daniele Contini, Angela Marinoni, Andres Alastuey, Tuukka Petäjä, Sergio Rodriguez, David Picard, Benjamin Brem, Max Priestman, David C. Green, David C. S. Beddows, Roy M. Harrison, Colin O’Dowd, Darius Ceburnis, Antti Hyvärinen, Bas Henzing, Suzanne Crumeyrolle, Jean-Philippe Putaud, Paolo Laj, Kay Weinhold, Kristina Plauškaitė, Steigvilė Byčenkienė

**Affiliations:** 1 Institute of Epidemiology, Helmholtz Zentrum München—German Research Center for Environmental Health, Neuherberg, Germany; 2 Environmental Science Center, University of Augsburg, Augsburg, Germany; 3 Experimental Aerosol and Cloud Microphysics, Leibniz Institute for Tropospheric Research, Leipzig, Germany; 4 The Lisbon Council, Brussels, Belgium; 5 Institute for Medical Research and Occupational Health, Zagreb, Croatia; 6 Andalusian Institute for Earth System Research (IISTA-CEAMA), University of Granada, Granada, Spain; 7 ANDRA – DISTEC-EES, Observatoire Pérenne de l’Environnement, Bure, France; 8 Climate and Atmosphere Research Center (CARE-C), The Cyprus Institute, Nicosia, Cyprus; 9 Department of Aerosol Chemistry and Physics, Institute of Chemical Process Fundamentals, CAS, Prague, Czech Republic; 10 Department of Atmospheric Sciences, University of Utah, Salt Lake City, USA; 11 Department of Environment, CIEMAT, Madrid, Spain; 12 Department of Physics, University of Malta, Msida, Malta; 13 Division of Physics, Division of Combustion Physics, Lund University, Lund, Sweden; 14 El Arenosillo - Atmospheric Sounding Station, Atmospheric Research and Instrumentation Branch, INTA, Mazagón, Huelva Spain; 15 Environmental Chemical Processes Laboratory, Department of Chemistry, University of Crete, Heraklion, Greece; 16 Institute for Environmental Research & Sustainable Development, National Observatory of Athens, I. Metaxa & Vas. Pavlou, Palea Penteli, Greece; 17 Department of Epidemiology, Institute for Medical Information Processing, Biometry, and Epidemiology, Ludwig-Maximilians-University Munich, Munich, Germany; 18 Centre for Cardiovascular Research (DZHK), Partner Site Munich Heart Alliance, Munich, Germany; 19 Environmental Radioactivity & Aerosol Tech. for Atmospheric & Climate Impacts, INRaSTES, National Centre of Scientific Research “Demokritos”, Paraskevi, Greece; 20 Forecast Research Division, National Institute of Meterological Sciences (NIMS), Seogwipo, Korea; 21 German Environment Agency, Berlin, Germany; 22 Institut Scientifique de Service Public (ISSeP), Liege, Belgium; 23 Institute for Nuclear Research and Nuclear Energy, Bulgarian Academy of Sciences, Sofia, Bulgaria; 24 Institute of Atmospheric Sciences and Climate (ISAC-CNR), Lecce, Italy; 25 Institute of Atmospheric Sciences and Climate, ISAC, Bologna, Italy; 26 Institute of Environmental Assessment and Water Research (IDAEA-CSIC), Barcelona, Spain; 27 Institute for Atmospheric and Earth System Research (INAR), Faculty of Science, University of Helsinki, Helsinki, Finland; 28 Izaña Atmospheric Research Centre, Agencia Estatal de Meteorología, Santa Cruz de Tenerife, Spain Group of Atmosphere, Aerosols and Climate-AAC, IPNA CSIC, Tenerife, Spain; 29 Laboratoire de Physique de Clermont Auvergne (LPCA), UMR6533, CNRS-UCA, Aubière, France; 30 Laboratory of Atmospheric Chemistry, Paul Scherrer Institute, Villigen PSI, Switzerland; 31 MRC Centre for Environment and Health, Environmental Research Group, Imperial College London, London, United Kingdom; 32 NIHR HPRU in Environmental Exposures and Health, Imperial College London, London, United Kingdom; 33 National Centre for Atmospheric Science, School of Geography, Earth and Environmental Sciences, University of Birmingham, Edgbaston, United Kingdom; 34 Department of Environmental Sciences, Faculty of Meteorology, Environment and Arid Land Agriculture, King Abdulaziz University, Jeddah, Saudi Arabia; 35 School of Natural Sciences, Ryan Institute’s Centre for Climate & Air Pollution Studies, University of Galway, Galway, Ireland; 36 SIOS Knowledge Centre, Svalbard science centre Longyearbyen, Longyearbyen, Norway; 37 The Netherlands Institute of Applied Scientific Research (TNO), Utrecht, Netherlands; 38 Univ. Lille, CNRS, UMR 8518 Laboratoire d’Optique Atmosphérique (LOA), Lille, France; 39 European Commission, Joint Research Centre, Ispra, Italy; 40 Univ. Grenoble, CNRS, IRD, IGE, Grenoble, France; 41 Center for Physical Sciences and Technology (FTMC), Vilnius, Lithuania

**Keywords:** Atmospheric dynamics, Atmospheric chemistry

## Abstract

Atmospheric new particle formation (NPF) is a naturally occurring phenomenon, during which high concentrations of sub-10 nm particles are created through gas to particle conversion. The NPF is observed in multiple environments around the world. Although it has observable influence onto annual total and ultrafine particle number concentrations (PNC and UFP, respectively), only limited epidemiological studies have investigated whether these particles are associated with adverse health effects. One plausible reason for this limitation may be related to the absence of NPF identifiers available in UFP and PNC data sets. Until recently, the regional NPF events were usually identified manually from particle number size distribution contour plots. Identification of NPF across multi-annual and multiple station data sets remained a tedious task. In this work, we introduce a regional NPF identifier, created using an automated, machine learning based algorithm. The regional NPF event tag was created for 65 measurement sites globally, covering the period from 1996 to 2023. The discussed data set can be used in future studies related to regional NPF.

## Background & Summary

Exposure to increased ultrafine particle number concentration (ultrafine particles, UFP, diameter <0.1 µm) poses a significant health risk^1–4^. Although several studies have reported a positive association between UFP exposure and increased adverse health-effects, the inconsistencies in epidemiological studies caused by not-harmonized UFP measurements, high spatial and temporal UFP variability, complex physical-chemical properties, etc., result in inconsistent findings regarding UFP impacts on health^5,6^. Moreover, UFP provides - through aerosol dynamic processes, a source for bigger particles that contribute to atmospheric light scattering and absorption, as well as the formation of cloud condensation nuclei^7^.

Unlike PM_2.5_ and PM_10_ (airborne particulate matter, with aerodynamic diameters ≤ 2.5 and 10 μm), the UFP is neither legally regulated nor consistently measured in long-term, official air quality monitoring sites. With that being said, particle number size distribution (PNSD) data does exist and was already used by several studies to report long-term European trends of UFP and total particle number (PNC) concentrations (e.g.^8–11^). The PNSD data are provided by Research Infrastructures (ACTRIS), international and European networks (GAW, EMEP) and regional research networks (German Ultrafine Aerosol Network, GUAN^12^,; the Spanish Network of Environmental Differential Mobility Analysers, REDMAAS^13^), Data repository for ACTRIS, GAW, EMEP and GUAN is hosted by EBAS@NILU, EBAS home – ebas homepage (nilu.no). The main origins of PNC and UFP particles in urban environments include but are not limited to road, sea and air traffic (e.g.^14–16^) emissions, long-range transport (e.g. Seto *et al*.^17^), emissions from residential heating and cooking (e.g.^18,19^), and new particle formation (NPF^20^). New particle formation and subsequent particle growth, which extend over a period of several days and forms a banana-shaped structure in the daily PNSD contour plots represents a regional phenomenon, which takes place over a large territory^21^. Other types of NPF (based on contour-plot shapes) were also observed, including bump- or apple-type structures^22,23^, which indicates a more local and/or disturbed nucleation. The NPF events have also been identified in urban environments (e.g.^24,25^), although such events are easier recognizable in more stable background environments^26^. In the past, the detection of regional NPF events relied on visual inspection of PNSDs (looking for a signature banana shape in a PNSD contour plots) and some automatic algorithms (^27,28^ and references therein). With an increasing availability and accessibility of deep learning or other novel machine learning methods in data analysis, such algorithms were also applied to classify between NPF event and non-event days^28,29^. Some studies have also discussed the NPF mechanisms, precursors, growth, and formation rates based on multi-year and multi-station data, automatically identifying nucleation events^30^.

During a regional NPF event, the urban background PNC and UFP number concentration (in a range from 10^3^ to 10^4^ cm^−3^) may suddenly increase up to an order of magnitude reaching levels like those observed in traffic impacted areas^10,31,32^. While people living at bigger distance to congested streets may experience a lower exposure to road-traffic emitted particles, regional NPF will undoubtedly lead to an increased exposure to PNC and UFP without the need for a traffic source nearby. Regional NPF has been shown to take place simultaneously over an area of up to several hundred kilometres. However, to date, only limited number of epidemiological studies (e.g.^33^) and to limited extent has included NPF events into data analysis. It remains uncertain, whether exposure to high PNC and UFP concentrations from regional nucleation, poses any health risks. The main reason for this may be the lack of a regional NPF identifier in long-term data set of PNC and UFP number concentrations. Furthermore, for health-related studies there is a clear need to separate UFP originating from combustion sources versus those being formed by regional atmospheric NPF events.

The main goal of this work is to provide the scientific community a regional NPF event identifier that can be used in future epidemiological studies to investigate the health-effects of PNC and UFP, based on a long-term (over 10 years) and global coverage data. The NPF classification is done by training a machine learning model to automatically detect regional NPF events. We focus on regional NPF events, specifically banana-shaped structures, because they have a broader impact on regional PNC and affect larger areas, thereby influencing populations far from measurement sites. In contrast, bump-type or apple-type NPFs are more localized and have a limited spatial extent. Additionally, accurately identifying non-regional NPFs requires complex labelling and additional data, which could introduce significant uncertainties. This way, our work provides, for the first time, a means to account for the regional NPF influence on long-term PNC and UFP concentration levels.

## Methods

**Fig. 1:**
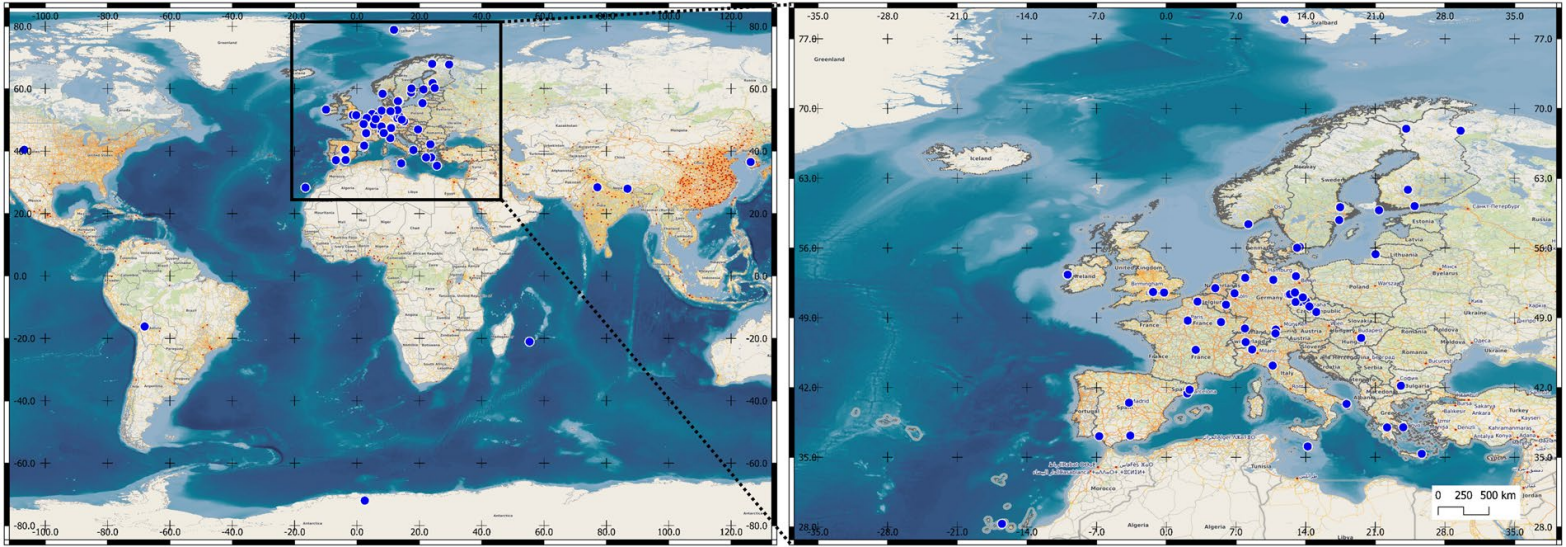
The global coverage of particle number size distribution measurement sites, retrieved from NILU EBAS.

Measurement sites, data availability, and preprocessing. The long-term global PNSD data on request (inquiring for specific period and spatial coverage) was received from the Norwegian Institute for Air Research (NILU) and EBAS@NILU in hourly resolution text-based NASA-Ames format. The PNSD data can also be freely downloaded from the NILU data base (https://ebas.nilu.no/). Downloading same data set would require the following steps: a) navigating to https://ebas-data.nilu.no; b) choosing country and station of interest; c) from a component field, choosing “particle number size distribution”; d) from a matrix field selecting “all”; e) and clicking list datasets. In the new page, one can identify period of interest and download required PNSD data. Finally, PNSD data can be obtained from each data originator directly. However, this would require additional effort compared to the first and second methods.; b) choosing country and station of interest;

The primary advantages of retrieving global PNSD data from the NILU database are the rigorous quality control and assurance measures and the uniform data format. The NILU database ensures that PNSD measurements, performed using mobility particle size spectrometers (MPSS), adhere to the well-defined standard operating procedures outlined in Wiedensohler *et al*.^34^. Each PNSD data file downloaded from the NILU database includes detailed header information, which encompasses the inlet type, humidity/temperature control, detection limits, measurement uncertainties, and various data tags, among other elements. Moreover, the EBAS@NILU database features a three-level data structure (level-0, level-1, and level-2), allowing end users to select the data structure that best suits their needs. In this study, we utilized level-2 data, which represent the final PNSD. These data are corrected to standard conditions of temperature (273.15 K) and pressure (1013.25 hPa) and are averaged to a time resolution of one hour. If required, users can also obtain level-0 (data set contains the metadata, raw data, and system parameters) and level-1 (data set contains processed (multiple charge and losses correction) PNSD with the original time resolution) data. The spatial coverage of the data is shown in Fig. 1.

**Fig. 2:**
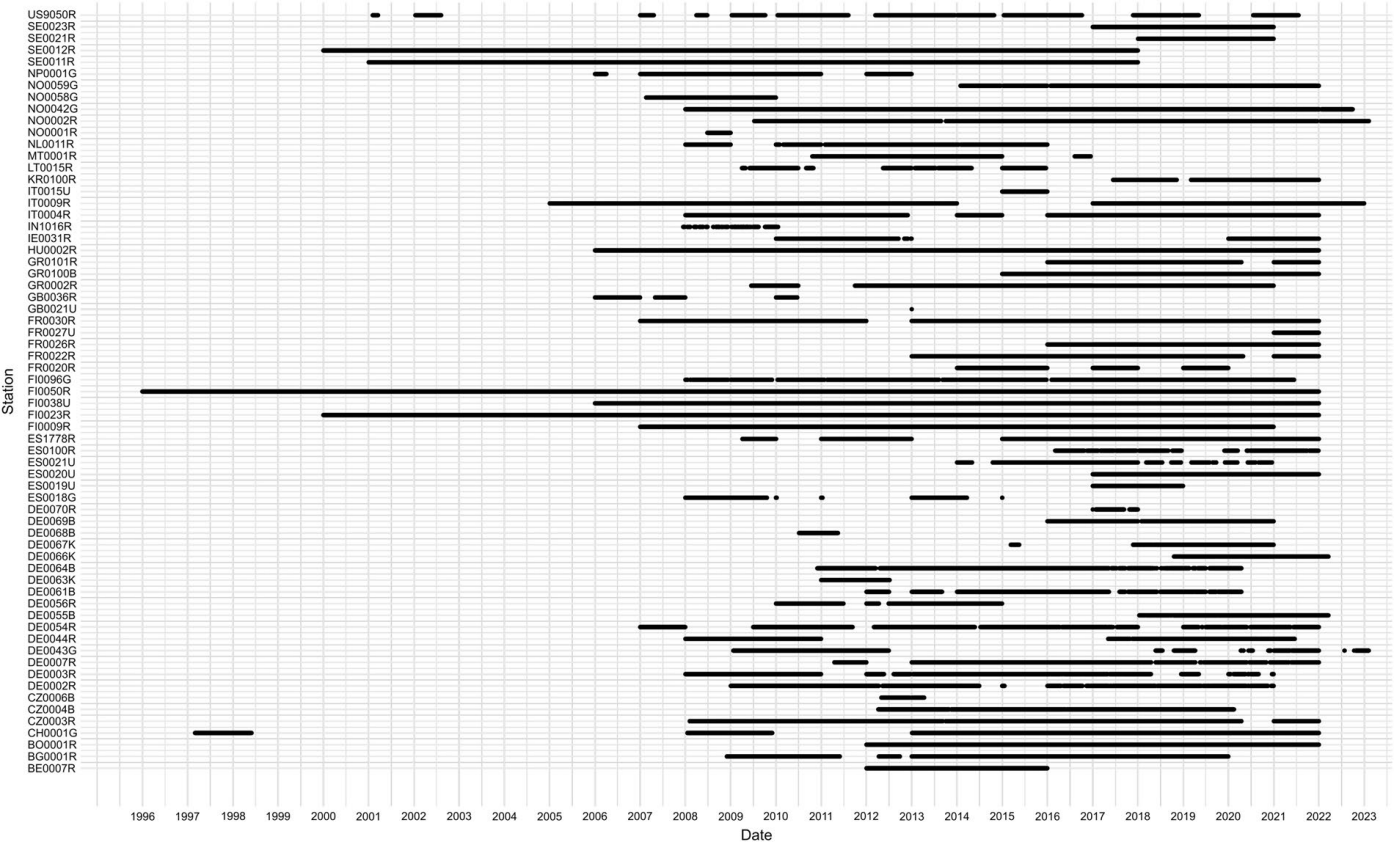
Data availability plot of the NPF event identifier.

Although the retrieved PNSD dataset was in a level-2 structure, indicating a harmonized data format, we observed discrepancies in the size ranges used for PNSD measurements across some of the measurement sites. The lowest reported diameter was 3 nm, and the highest - 1357 nm. In addition, the measured particle size range varied across different measurement sites, including ranges from 8.82 to 333.8 nm, 8.82 to 289 nm, 6.3 to 389.31 nm, and 3.16 to 1000 nm, among others. Such discrepancies between sites, although have no effect on identifying regional NPF events, it make comparison between PNSD derived parameters (e.g., integrated particle number concentration, particle formation and growth rates, etc.) rather difficult. Besides different size ranges, further differences between received data files were observed: (1) not all station data was of level-2 (in some instances, other levels were identified in a requested level-2 data file names). In this study, we used the highest level data. That is, if level-2 data was not available, level-1.5 data was used with no specific treatment. It must be noted that regional NPF identification is possible using either level data (because banana shape structure would appear in any level data contour plot); (2) some data files included only one diameter (and not a diameter range). Such data was excluded from further analysis; (3) some data files had a different structure; (4) missing data was identified differently (e.g. 999.9999, 99.99, 9.9, etc.). Although the standard data format required by the database implies unity between different measurement sites, some of the issues listed above greatly increased the effort to automatically reprocess the data. All the PNSD measurement sites, used for regional NPF event classification are listed in Tables 1–3. The station type classification is partly based on Rose *et al*.^10^ and the site description in level-2 PNSD files. The NPF event classification covers many environments including rural background, forest, urban, suburban, mountain, pristine, and mixed. Temporal coverage of the NPF event identifier is show in Fig. 2.

**Table 1:** The measurement sites used for regional NPF event classification.

Nr.	Station Code	Station Name	Lat.	Long.	Alt.	Type	Bad (N = 1034)	Non-NPF (N = 2777)	NPF (N = 1008)
1	KR0100R	Anmyeon-do	36.538	126.330	46	RB, Coast	+	+	+
2	DE0061B	Annaberg-Buchholz	50.571	12.998	545	U	+	—	—
3	SE0012R	Aspvreten	58.800	17.383	20	F	+	—	—
4	ES0019U	Barcelona	41.390	2.116	80	U	+	+	+
5	BG0001R	BEO Moussala	42.166	23.583	2971	M	+	+	+
6	NO0002R	Birkenes II	58.388	8.252	219	F	—	+	—
7	NL0011R	Cabauw Zijdeweg	51.970	4.926	1	RB	+	—	—
8	GR0100B	Demokritos Athens	37.994	23.815	270	S, Coast	+	+	+
9	DE0070R	Deutschneudorf	50.603	13.465	660	U	—	—	+
10	DE0063K	Dresden-Nord	51.065	13.741	116	U	+	+	+
11	DE0064B	Dresden-Winckelmannstrasse	51.036	13.730	120	U	—	+	+
12	ES0100R	El Arenosillo	37.100	−6.733	41	F	+	+	+
13	GR0002R	Finokalia	35.337	25.669	250	RB, Coast	—	+	+
14	MT0001R	Giordan Lighthouse	36.072	14.218	167	RB	+	+	+
15	ES0020U	Granada	37.164	−3.605	680	U	+	—	—
16	IN1016R	Gual Pahari	28.427	77.151	320	U	+	—	—
17	GB0036R	Harwell	51.573	−1.316	137	U	—	+	+
18	GR0101R	Helmos Mountain	37.984	22.196	2340	M	+	—	+
19	DE0043G	Hohenpeissenberg	47.801	11.009	975	RB	+	—	+
20	SE0021R	Hyltemossa	56.097	13.418	115	F	—	+	+
21	FI0050R	Hyytiälä	61.850	24.283	181	F	—	+	+
22	IT0004R	Ispra	45.81	8.63	209	UB		+	+

The total number of daily contour plots, used for model training is shown in brackets. The country of origin can be read from the first two letters of the station code string. For a further description of measurement sites, please refer to Rose *et al*.^10^. The station surroundings are described by type, with RB = Rural background; U – Urban; F – Forest; M – Mountain; S – Suburban; P – Pristine. The “—” and “ + ” symbols indicate whether specific data was included (“—” not; “ + ” yes) in the machine learning model training. The word “Coast” is added to the sites, which are near the coastal line.

**Table 2:** Continuation of Table 1 - the measurement sites used for regional NPF event classification.

Nr.	Station Code	Station Name	Lat.	Long.	Alt.	Type	Bad (N = 1034)	Non-NPF (N = 2777)	NPF (N = 1008)
1	ES0018G	Izana	28.309	−16.499	2373	M	+	+	—
2	CH0001G	Jungfraujoch	46.547	7.985	3578	M	+	+	—
3	HU0002R	K-puszta	46.966	19.583	125	RB	+	—	—
4	CZ0003R	Kosetice (NAOK)	49.573	15.080	535	RB	+	+	+
5	FI0038U	Kumpula	60.202	24.961	25	U	+	—	—
6	FR0026R	La Réunion	−21.079	55.383	2160	M	+	+	+
7	DE0066K	Leipzig-Eisenbahnstrasse	51.345	12.406	120	U	—	—	+
8	DE0067K	Leipzig-Mitte	51.344	12.377	111	U	—	—	+
9	DE0068B	Leipzig-West	51.318	12.297	122	U	—	+	—
10	IE0031R	Mace Head	53.325	−9.899	5	RB, Coast	—	+	—
11	ES1778R	Montseny	41.767	2.350	700	RB	—	—	—
12	IT0009R	Monte Cimone	44.193	10.7014	2165	M	+	—	—
13	BO0001R	Mount Chacaltaya	−16.200	−68.099	5320	M	—	—	+
14	DE0069B	Mülheim-Styrum	51.453	6.865	39	S	—	+	—
15	NP0001G	Nepal Climate Observatory - Pyramid	27.957	86.814	5079	M	+	+	—
16	DE0007R	Neuglobsow	53.166	13.033	62	F	+	—	+
17	SE0023R	Norunda Tornet	60.086	17.479	46	F	—	—	+
18	FR0022R	Observatoire Perenne de l’Environnement	48.562	5.505	392	RB	+	+	+
19	FI0096G	Pallas	67.973	24.116	565	P	—	+	+
20	CZ0004B	Prague-Suchdol	50.126	14.385	270	U	—	—	—
21	LT0015R	Preila	55.376	21.030	5	RB, Coast	+	—	—
22	FR0030R	Puy de Dôme	45.772	2.964	1465	M	—	—	+

For the table explanation, please refer to Table 1.

**Table 3:** Continuation of Tables 1 and 2 - the measurement sites used for regional NPF event classification.

Nr.	Station Code	Station Name	Lat.	Long.	Alt.	Type	Bad (N = 1034)	Non-NPF (N = 2777)	NPF (N = 1008)
1	DE0003R	Schauinsland	47.914	7.908	1205	M	—	+	+
2	US9050R	Steamboat Springs	40.445	−106.740	3220	M	+	—	—
3	BE0007R	TMNT09 Vielsalm	50.304	6.001	496	F	—	+	—
4	NO0058G	Troll	−72.016	2.533	1309	M	+	—	—
5	CZ0006B	Ústí n.L.-mesto	50.661	14.040	147	U	—	—	+
6	FI0009R	Utö	59.779	21.377	7	RB	+	+	+
7	FI0023R	Värriö	67.766	29.583	400	RB	+	—	+
8	SE0011R	Vavihill	56.016	13.150	175	F	+	—	+
9	FR0027U	Villeneuve d’Ascq	50.611	3.140	70	U	—	+	—
10	DE0002R	Waldhof	52.802	10.759	74	F	—	+	+
11	NO0042G	Zeppelin mountain	78.907	11.886	474	M	+	+	—
12	DE0054R	Zugspitze-Schneefernerhaus	47.416	10.979	2671	M	—	—	—
13	NO0001R	Birkenes	58.380	8.250	190	F	—	—	—
14	DE0055B	Leipzig	51.352	12.434	113	U	—	—	—
15	ES0021U	Madrid	40.460	−3.730	669	U	—	—	—
16	NO0059G	Trollhaugen	−72.010	2.540	1553	M	—	—	—
17	DE0056R	Bösel	53.000	7.940	40	U	—	—	—
18	FR0020R	SIRTA	48.710	2.160	162	U	—	—	—
19	DE0044R	Melpitz	51.530	12.930	86	RB	—	—	—
20	IT0015U	Lecce	40.340	18.120	36	U	—	—	—
21	GB0021U	London - North Kensington	51.520	−0.210	27	U	—	—	—

For the table explanation, please refer to Table 1.

### Recognition of regional new particle formation events

Given that daily PNSD measurement data can be represented as a contour image, the convolutional neural networks (CNNs) were used for automatic classification of three different daily events categories: NPF event day, non-NPF event day, and bad data. The CNNs are a class of deep neural networks, mostly used with grid-like topology data, such as images. It utilizes the convolutional layers to learn spatial hierarchies of features but demand large complexity and rich data to extract relevant features^35^. Shortly, a CNNs works by (a) detecting patterns, (b) combining clues, (c) making decisions, and (d) learning from examples. The CNNs scans the image for specific patterns, like edges or textures, after which it pieces them together to understand the overall content of the image. By using the gathered information, the CNNs algorithm decides what the image represents, choosing from predefined categories. Through training with many examples, the CNN improves its ability to correctly classify images by adjusting its internal settings based on its successes and mistakes. As shown previously, the application of CNNs in image-like data processing is an effective way to identify NPF events from the contour plots^28^. The CNNs model used in this work is known as Microsoft Residual Network (ResNet^36^). The pretrained and publicly available ResNet model was loaded and applied to previously processed data using Google Colaboratory (accessed December 2023), Google Research, available from https://colab.research.google.com/) engined by Python 3.xx (www.python.org) Google Compute Engine backend (GPU with 12.7 GB of System RAM; 15.0 GB GPU RAM; and 78.2 GB of Disk space). The “fastai” and “PyTorch” packages were used for this purpose^37,38^. The model was trained using the following steps:

1. The PNSDs from NASA-Ames format files were extracted, and 1-hour time resolution contour plots (1 per day) were plotted using R statistical computing software (R Core Team).
2. Three categories were chosen for PNSD classification, namely – “bad data” (representing non-continuous PNSD, missing data, etc.), “non-NPF” (or regular), and “NPF”. Fewer or more classes can be used, however, in case of data usage for epidemiological studies, determining missing data (for filling in the gaps), and NPF versus non-NPF event days satisfied our aim.
3. Random cases with PNSDs representing bad, non-NPF, and NPF cases were then labelled by a skilled researcher. In total, 1034, 2777, and 1008 contour plots were selected to represent PNSD cases of bad, non-NPF, and NPF events, respectively. The exemplary PNSD can be seen in Fig. 3. All figures were labelled according to the case they represent. This step is vital for the training of the CNNs model. It must be noted that in this work we only consider regional NPF and subsequent particle growth, which forms easily recognizable banana shape in PNSD contour plot. This choice was made because a) regional NPF may influence PNC on larger spatial scale and thus be more relevant than locally occurring events; and b) it is not trivial to identify locally occurring bursts of new particles (only having PNSD information) and separate them from e.g. local traffic or other emissions.

**Fig. 3:**
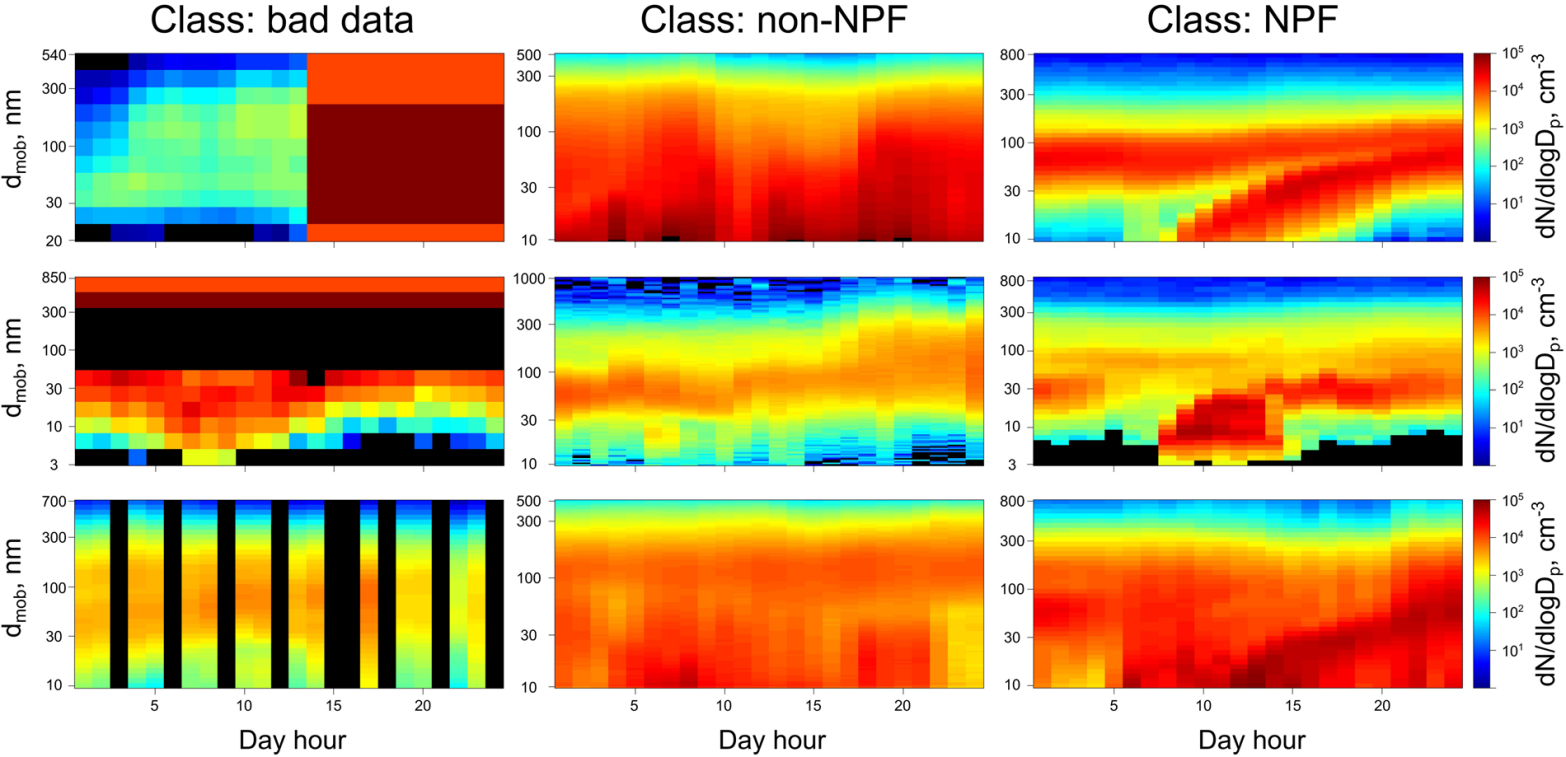
Exemplary cases for particle number size distribution contour plots, which were used for model training. Class “bad data” here refers to cases when PNSDs were non-continuous, missing data was present, etc.
4. The Residual Network with 50 layers (ResNet-50) was used for image classification. It was chosen because of its ability to address the challenge of vanishing gradients in deep networks by using residual or skip connections. These connections enable the network to learn residual functions, facilitating effective gradient propagation during training. ResNet-50’s 50-layer structure, consisting of convolutional, pooling, and fully connected layers, contributes to its ability to achieve state-of-the-art performance on various computer vision tasks, making it a valuable tool in the field of deep learning for image analysis. A fine tune parameter of 6 was used which indicates that during the fine-tuning process of ResNet-50 for image classification, only 6 specific layers (closer to the output) are being adjusted to better fit the new dataset.

## Data Records

The global NPF identifier, PNSDs used for model training, the trained CNNs model, and used codes files were deposited in Figshare under a DOI (Digital Object Identifier) of 10.6084/m9.figshare.25375978.v2^39^. The data set consists of 1 zipped folder, which contains 6 files. In the zipped folders, named PNSD_NPF, PNSD_nonNPF, and PNSD_BAD exemplary cases of particle number size distributions, used for model training, are presented. The CNNs model, trained on provided PNSDs is provided in file NPF_CNN_model. The classification of PNSDs, using trained CNNs model (NPF_CNN_model) is given in Kecorius_et_al_NPF_identifier file (in a format of Table 4). The Python codes, used in Google Colaboratory age given in file Google_Colab_Code.

**Table 4:** The example of NPF identifier data set.

Date	Station Code	TAG [NPF = 1; non-NPF = 0; bad = −1]	Prediction [%]
2007-04-07	FI0038U	1	91
2020-01-29	SE0021R	−1	100
2014-10-26	FR0030R	0	99
…	…	…	…

The provided data set format of the NPF identifier is shown in Table 4. The data set comprises of four columns, namely Date (year, month and day), Station Code (a unique station identifier, which can be used to retrieve its location based on the information in Tables 1–3), TAG (an identifier for NPF = 1, non-NPF = 0, and bad data = −1), and Prediction (the ML model probability score in percent). The prediction refers to the model’s confidence regarding its prediction. In the context of image classification, the CNNs produce a probability distribution over all classes after processing an image. Each class is assigned with a probability score between 0 and 1, representing the model’s confidence that the image belongs to that class. Higher percentages indicate higher confidence in the predicted class. It is worth noting that one may consider only predictions where the highest probability score exceeds a certain threshold as valid predictions. For example, based on Table 4, on 7 April 2007, a NPF event was registered at FI0038U measurement station in Finland with a 91% certainty. In general, the use of confidence scores from the prediction shall be based on the specific needs of the study. For example, if one desires to maximize the coverage of the dataset, prediction confidence between 75 and 100% may be chosen. In this case, the subset data would retain 90% of original data. On the other hand, if accuracy is preferred, we suggest using prediction confidence, which is greater than 90% (retaining 80% of original data). This threshold is based on empirical evaluation and cross-validation results, which indicate that predictions with confidence scores above 90% are associated with higher accuracy and lower uncertainty.

## Technical Validation

**Fig. 4:**
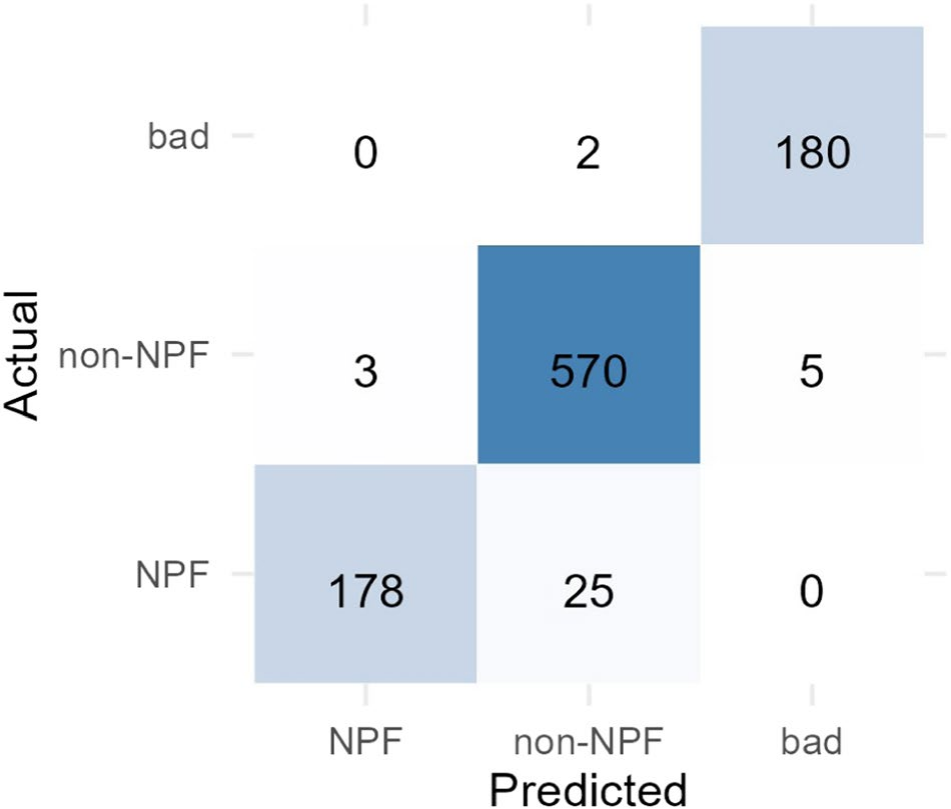
Confusion matrix for evaluation of ML model performance.

The CNNs model was trained and was evaluated with the data presented in Data Records section. The results are presented by means of a confusion matrix, which provides a detailed summary of the model’s predictions compared to the actual labels in the dataset (Fig. 4).

Based on the confusion matrix, two model performance metrics were calculated to judge the model’s accuracy – the area under the receiver operating characteristic curve (AUC) and the F1 score. Shortly, the Receiver Operating Characteristic (ROC) curve is a graphical representation of a binary identifier’s performance across various threshold settings. It plots the true positive rate (sensitivity) against the false positive rate (1-specificity) for different threshold values. The AUC quantifies the overall performance of the model across all thresholds. The AUC can be calculated based on the True Positive Rate (TPR) and False Positive Rate (FPR) across different threshold settings:

1 $${TPR}=\frac{{TP}}{\left({TP}+{FN}\right)},$$

2 $${FPR}=\frac{{FP}}{\left({FP}+{TN}\right)},$$

where TP are true positives, FN are false negatives, FP are false positives, and TN are true negatives. The AUC calculation was performed by plotting TPR against FPR at various threshold settings and computing the area under this curve. AUC ranges from 0 to 1, where 0 indicates deficient performance (the identifier always predicts the wrong class) and 1 indicates perfect performance (the identifier always predicts the correct class). AUC provides a single scalar value representing the model’s ability to discriminate between positive and negative classes. The F1 score is a metric that combines both precision and recall into a single value. The F1 score is the harmonic mean of precision (the proportion of true positive predictions among all positive predictions made by the model) and recall (the proportion of true positive predictions among all actual positive samples). It can be calculated as:

3 $${Precision}\left(P\right)=\frac{{TP}}{\left({TP}+{FP}\right)},$$

4 $${Recall}\left(R\right)=\frac{{TP}}{\left({TP}+{FN}\right)},$$

5 $$F1=2\times \frac{\left(P\times R\right)}{\left(P+R\right)}.$$

The F1 score reaches its best value at 1 and its worst value at 0. It provides a balance between precision and recall. In the context of evaluating CNNs, Eqs. 1–5 are applied by considering the predictions made by the model and comparing them against the ground truth labels of the dataset. The TP, TN, FP, and FN are counted based on the model’s predictions and the actual labels. For our model, the AUC and F1 score are 0.99 and 0.93, respectively, indicating a satisfactory model performance.

## Data Availability

The custom Python codes, used to train the CNNs model in Google Colaboratory is freely available at 10.6084/m9.figshare.25375978.v2^39^. Statistical analysis and plotting were performed using the open-source programming language and software environment R (R Core Team, 2013; version 4.2.2)^40^. For spatial data representation, a quantum geographic information system (QGIS Development Team, 2022)^41^ was used.

## References

[1] Chen, R. *et al*. Beyond PM2. 5: The role of ultrafine particles on adverse health effects of air pollution. *Biochimica et Biophysica Acta (BBA)-General Subjects* **1860**(12), 2844–2855 (2016). 26993200 10.1016/j.bbagen.2016.03.019

[2] Kwon, H. S., Ryu, M. H. & Carlsten, C. Ultrafine particles: unique physicochemical properties relevant to health and disease. *Experimental & molecular medicine* **52**(3), 318–328 (2020). 32203103 10.1038/s12276-020-0405-1 PMC7156720

[3] Peters, A., Wichmann, H. E., Tuch, T., Heinrich, J. & Heyder, J. Respiratory effects are associated with the number of ultrafine particles. *American journal of respiratory and critical care medicine* **155**(4), 1376–1383 (1997). 9105082 10.1164/ajrccm.155.4.9105082

[4] Schwarz, M. *et al*. Impact of ultrafine particles and total particle number concentration on five cause-specific hospital admission endpoints in three German cities. *Environment International* **178**, 108032 (2023). 37352580 10.1016/j.envint.2023.108032

[5] Abdillah, S. F. & Wang, Y. F. Ambient ultrafine particle (PM0.1): Sources, characteristics, measurements and exposure implications on human health. *Environmental Research* **218**, 115061 (2023). 36525995 10.1016/j.envres.2022.115061

[6] Cassee, F. *et al*. 2019. White Paper: Ambient ultrafine particles: evidence for policy makers.

[7] Kerminen, V.-M. *et al*. Cloud condensation nuclei production associated with atmospheric nucleation: a synthesis based on existing literature and new results. *Atmos. Chem. Phys.* **12**, 12037–12059, 10.5194/acp-12-12037-2012 (2012).

[8] Garcia-Marlès, M. *et al*. Inter-annual trends of ultrafine particles in urban Europe. *Environment international* **185**, 108510 (2024). 38460241 10.1016/j.envint.2024.108510

[9] Liu, X. *et al*. Ambient air particulate total lung deposited surface area (LDSA) levels in urban Europe. *Science of the Total Environment* **898**, 165466 (2023). 37451445 10.1016/j.scitotenv.2023.165466

[10] Rose, C. *et al*. Seasonality of the particle number concentration and size distribution: a global analysis retrieved from the network of Global Atmosphere Watch (GAW) near-surface observatories. *Atmospheric Chemistry and Physics Discussions* **2021**, 1–69 (2021).

[11] Savadkoohi, M. *et al*. The variability of mass concentrations and source apportionment analysis of equivalent black carbon across urban Europe. *Environment international* **178**, 108081 (2023). 37451041 10.1016/j.envint.2023.108081

[12] Birmili, W. *et al*. Long-term observations of tropospheric particle number size distributions and equivalent black carbon mass concentrations in the German Ultrafine Aerosol Network (GUAN). *Earth System Science Data*, p.355 (2016).

[13] Alonso-Blanco, E. *et al*. Temporal and spatial variability of atmospheric particle number size distributions across Spain. *Atmospheric environment* **190**, 146–160 (2018).

[14] Hopke, P. K., Feng, Y. & Dai, Q. Source apportionment of particle number concentrations: A global review. *Science of the Total Environment* **819**, 153104 (2022). 35038523 10.1016/j.scitotenv.2022.153104

[15] Lopes, M., Russo, A., Monjardino, J., Gouveia, C. & Ferreira, F. Monitoring of ultrafine particles in the surrounding urban area of a civilian airport. *Atmospheric Pollution Research* **10**(5), 1454–1463 (2019a).

[16] Lopes, M., Russo, A., Gouveia, C. & Ferreira, F. Monitoring of ultrafine particles in the surrounding urban area of in-land passenger ferries. *Journal of Environmental Protection* **10**(06), 838 (2019b).

[17] Seto, T. *et al*. New particle formation and growth associated with East-Asian long-range transportation observed at Fukue Island, Japan in March 2012. *Atmospheric Environment* **74**, 29–36 (2013).

[18] Wallace, L. & Ott, W. Personal exposure to ultrafine particles. *Journal of exposure science & environmental epidemiology* **21**(1), 20–30 (2011). 20087407 10.1038/jes.2009.59

[19] Wang, D. *et al*. Significant ultrafine particle emissions from residential solid fuel combustion. *Science of The Total Environment* **715**, 136992 (2020). 32023515 10.1016/j.scitotenv.2020.136992

[20] Kulmala, M. *et al*. Formation and growth rates of ultrafine atmospheric particles: a review of observations. *Journal of Aerosol Science* **35**(2), 143–176 (2004).

[21] Ström, J., Engvall, A. C., Delbart, F., Krejci, R. & Treffeisen, R. On small particles in the Arctic summer boundary layer: observations at two different heights near Ny-Ålesund, Svalbard. *Tellus B: Chemical and Physical Meteorology* **61**(2), 473–482 (2009).

[22] Ehn, M. *et al*. 2010. Growth rates during coastal and marine new particle formation in western Ireland. *Journal of Geophysical Research: Atmospheres*, 115(D18).

[23] Vana, M. *et al*. Characteristic features of air ions at Mace Head on the west coast of Ireland. *Atmospheric Research* **90**(2–4), 278–286 (2008).

[24] Hofman, J. *et al*. Ultrafine particles in four European urban environments: Results from a new continuous long-term monitoring network. *Atmospheric environment* **136**, 68–81 (2016).

[25] Hussein, T. *et al*. Observation of regional new particle formation in the urban atmosphere. *Tellus B: Chemical and Physical Meteorology* **60**(4), 509–521 (2008).

[26] Kulmala, M. *et al*. Quiet new particle formation in the atmosphere. *Frontiers in Environmental Science* **10**, 912385 (2022).

[27] Größ, J. *et al*. Atmospheric new particle formation at the research station Melpitz, Germany: connection with gaseous precursors and meteorological parameters. *Atmos. Chem. Phys.* **18**, 1835–1861, 10.5194/acp-18-1835-2018 (2018).

[28] Joutsensaari, J. *et al*. Identification of new particle formation events with deep learning. *Atmospheric Chemistry and Physics* **18**(13), 9597–9615 (2018).

[29] Su, P. *et al*. New particle formation event detection with Mask R-CNN. *Atmospheric Chemistry and Physics* **22**(2), 1293–1309 (2022).

[30] Dall’Osto, M. *et al*. Novel insights on new particle formation derived from a pan-european observing system. *Scientific reports* **8**(1), 1482 (2018). 29367716 10.1038/s41598-017-17343-9 PMC5784154

[31] Pushpawela, B., Jayaratne, R. & Morawska, L. Temporal distribution and other characteristics of new particle formation events in an urban environment. *Environmental Pollution* **233**, 552–560 (2018). 29102885 10.1016/j.envpol.2017.10.102

[32] Wang, Z. B. *et al*. Characteristics of regional new particle formation in urban and regional background environments in the North China Plain. *Atmospheric Chemistry and Physics* **13**(24), 12495–12506 (2013).

[33] Rivas, I. *et al*. Associations between sources of particle number and mortality in four European cities. *Environment International* **155**, 106662 (2021). 34098335 10.1016/j.envint.2021.106662

[34] Wiedensohler, A. *et al*. Mobility particle size spectrometers: harmonization of technical standards and data structure to facilitate high quality long-term observations of atmospheric particle number size distributions. *Atmospheric Measurement Techniques* **5**(3), 657–685 (2012).

[35] LeCun, Y., Bengio, Y. & Hinton, G. Deep learning. *nature* **521**(7553), 436–444 (2015). 26017442 10.1038/nature14539

[36] He, K., Zhang, X., Ren, S. & Sun, J. Deep residual learning for image recognition. In *Proceedings of the IEEE conference on computer vision and pattern recognition* pp. 770–778 (2016).

[37] Howard, J. & Gugger, S. Deep Learning for Coders with fastai and PyTorch. O’Reilly Media (2020a).

[38] Howard, J. & Gugger, S. Fastai: a layered API for deep learning. *Information* **11**(2), 108 (2020b).

[39] Kecorius, S. *et al*. Atmospheric new particle formation identifier using longitudinal global particle number size distribution data figshare 10.6084/m9.figshare.25375978.v2 (2024). 10.1038/s41597-024-04079-1 PMC11569151 39550387

[40] R Core Team. R: A language and environment for statistical computing, R Foundation for Statistical Computing, Vienna, Austria (2013).

[41] QGIS Development Team. QGIS Geographic Information System. Open Source Geospatial Foundation Project. Available at: https://qgis.org [Accessed: 3 April 2023] (2022).

